# Optimizing Care for Primary Glomerulonephritis: The Role of Thyroid Evaluation

**DOI:** 10.2174/0118715303328436241121114054

**Published:** 2025-01-08

**Authors:** Ahmet Numan Demir, Cebrail Karaca, Zehra Kara, Cem Sulu, Serdar Sahin, Emre Durcan, Mevlut Tamer Dincer, Sukran Erdem Nurcan, Ozge Sonmez, Hande Mefkure Ozkaya, Nurhan Seyahi, Mustafa Sait Gonen

**Affiliations:** 1 Department of Endocrinology, Metabolism, and Diabetes, Istanbul University-Cerrahpasa, Istanbul, Turkey;; 2 Department of Nephrology, Istanbul University-Cerrahpasa, Istanbul, Turkey;; 3 Department of Internal Medicine, Istanbul University-Cerrahpasa, Istanbul, Turkey

**Keywords:** Nodular goiter, primary glomerulonephritis, autoimmune thyroid disease, glomerulonephritis, thyroid ultrasonography, hypothyroidism, thyroid disorders

## Abstract

**Background:**

The coexistence of primary glomerulonephritis and autoimmune thyroid disease has not been investigated.

**Objective:**

This study aimed to assess thyroid morphology using sonography, determine the prevalence of autoimmune thyroid disorders, and evaluate thyroid function status in patients diagnosed with primary glomerulonephritis.

**Materials and Methods:**

This single-center cross-sectional and observational study included 58 consecutive patients with primary glomerulonephritis and 58 healthy controls (HC). All participants underwent thyroid examination through laboratory tests and thyroid ultrasonography. The findings were subsequently compared between the two groups.

**Results:**

Among the patients, 17.2% (*n* = 10) exhibited subclinical hypothyroidism, while 8.6% (*n* = 5) had overt hypothyroidism. None of the HCs showed overt hypothyroidism, whereas 3.4% (*n* = 2) of them exhibited subclinical hypothyroidism. Patients displayed significantly lower free triiodothyronine (FT3) levels than HCs (*p* < 0.001). A linear correlation was observed between patients' thyroid-stimulating hormone (TSH) levels and the degree of proteinuria (*p* = 0.044). Furthermore, thyroid volume (*p* < 0.001), hypoechogenicity (*p* < 0.001), heterogeneous structure (*p* < 0.001), pseudonodular hypoechoic infiltration (*p* = 0.05), and the presence of nodules (*p* < 0.001) were notably higher in patients compared to HCs.

**Conclusion:**

The prevalence of both overt and subclinical hypothyroidism, along with the frequency of nodular goiter, was found to be elevated in patients with primary glomerulonephritis. Considering that primary glomerulonephritis predominantly afflicts young individuals, and these patients bear the lifelong burden of chronic kidney disease, we underscore the significance of routine thyroid function tests and sonographic examinations for the early detection of thyroid disorders.

## 1 INTRODUCTION

Primary glomerulonephritis is a rare disease that mainly affects young people and is the most common cause of end-stage renal disease in this population [1]. The clinical course of glomerulonephritis is highly variable, ranging from asymptomatic cases characterized by incidental findings, such as hypertension, proteinuria, hematuria, and elevated serum creatinine concentrations, to rapidly progressive glomerulonephritis associated with excessive weight gain and edema, culminating in uremia in nephrotic syndrome [2]. An essential aspect in assessing suspected cases is the distinction between primary and secondary glomerulonephritis, as the latter often requires treatment of the underlying disease rather than the glomerulonephritis itself [3]. In contrast, there are usually no specific treatment options for primary glomerulonephritis, making it a lifelong burden of disease [4].

Recent studies have reported an increased risk of developing chronic glomerulonephritis in individuals with autoimmune thyroid disease [5]. However, there is little literature on autoimmune thyroid disease in glomerulonephritis patients. Previous research has mainly focused on people with chronic kidney disease and end-stage renal disease, in whom endocrine disorders, including thyroid dysfunction, are prevalent [6]. Primary hypothyroidism (non-autoimmune) is frequently observed in patients with chronic kidney disease (CKD). In particular, the prevalence of subclinical hypothyroidism tends to increase when the glomerular filtration rate (GFR) decreases [7]. In addition, structural thyroid abnormalities are more common in people with CKD than in the general population, such as goiter, thyroid nodules, and thyroid carcinoma [8].

Considering this, this study aims to comprehensively assess thyroid morphology through sonography, determine the prevalence of autoimmune thyroid disease, and evaluate thyroid function status in patients with primary glomerulonephritis.

## 2 MATERIALS AND METHODS

### 2.1 Study Design

This cross-sectional study was conducted in the Department of Endocrinology and Nephrology at a tertiary care university hospital. It was an observational study conducted using STROBE guidelines.

### 2.2 Procedure

Patients with primary glomerulonephritis were selected consecutively from patients treated at the Department of Nephrology between 2020 and 2022. The diagnosis of primary glomerulonephritis was based on laboratory tests, clinical signs, symptoms, and a kidney biopsy. The inclusion criteria were: (i) age over 18 years, (ii) a pathologically confirmed diagnosis of primary glomerulonephritis. Exclusion criteria included: (i) a previous diagnosis of thyroid disease, (ii) active malignant disease, (iii) thyroidectomy, (iv) radiotherapy to the neck, (v) radioiodine therapy, (vi) use of medications, such as amiodarone, and (vii) recent contrast administration within the last six months. Healthy control subjects were selected from volunteers who had registered for routine medical examinations for applications between November, 2020 and December, 2021. Thyroid findings were then compared between patients with primary glomerulonephritis and healthy controls.

### 2.3 Data and Sample Collection

The patients underwent a comprehensive examination, including physical examinations, laboratory tests, and thyroid sonography. Biometric data, such as age, sex, height, weight, blood pressure, and pulse rate, were recorded in detail. Biochemical parameters collected under fasting conditions included thyroid stimulating hormone (TSH), free triiodothyronine (FT3), free thyroxine (FT4), thyroglobulin antibody (anti-TG), thyroid peroxidase antibody (anti-TPO), uric acid, total cholesterol, low-density lipoprotein (LDL), triglycerides (TG), high-density lipoprotein (HDL), and C-reactive protein (CRP), which were taken from the medical records.

The thyroid-stimulating hormone was quantified by the sandwich method using two different monoclonal antibodies labeled with biotin or a ruthenium complex. FT3 and FT4 were measured using a competitive technique, a specific monoclonal antibody labeled with a ruthenium complex, and the electrochemiluminescence immunoassay (ECLIA) method. These parameters were analyzed using the Elecsys test kits on the Roche Cobas e 602 systems. In addition, anti-TPO and anti-TG levels were determined by a competitive procedure using the ECLIA method. The enzymatic colorimetric method quantified uric acid, total cholesterol, LDL, TG, and HDL. C-reactive protein levels were determined using the immunoturbidimetric method. All these parameters were analyzed using the Roche/Hitachi Cobas c systems (Roche Diagnostics GmbH, Mannheim, Germany).

### 2.4 Clinical Definition

The FT3 and FT4 values were within the normal reference range, while TSH values above the reference range indicated subclinical hypothyroidism. If both FT3 and FT4 were below the reference range while TSH values were above the reference range, overt hypothyroidism was diagnosed. Anti-TG and anti-TPO levels in serum were considered positive if they exceeded 115 IU/mL and 34 IU/mL, respectively. If anti-TPO and anti-TG levels were positive, a diagnosis of Hashimoto's thyroiditis was made [9-11].

### 2.5 Sonography and VESINC System

Patients were instructed to hyperextend their necks according to a standard ultrasound protocol. Each thyroid lobe was scanned separately in the axial, longitudinal, and transverse planes. This was performed by the same physician using a LOGIQ e ultrasound machine (software version R9.1.2; General Electric Company, China, 2016) equipped with a 6–12 MHz linear transducer. The thyroid volume was calculated using the ellipsoid formula: Volume (ml) = Depth (cm) x Width (cm) x Length (cm) x π/6 [12]. An average thyroid volume was defined as 10 mL [13].

The structure of the parenchyma was assessed according to the VESINC sonographic classification system [14, 15]. The VESINC parameters include: V, volume in cubic centimeters (mL); E, echogenicity (E1, isoechoic; E2, mildly hypoechoic or E3, hypoechoic); S, sonographic texture (S1, homogeneous or S2, heterogeneous); I, pseudonodular hypoechoic infiltration (I0, absent or I1, present); N, nodules (N0, absent or N1, present); C, cysts (C0, absent or C1, present).

### 2.6 Statistical Analysis

The statistical analyses used the Statistical Package for the Social Sciences (SPSS) software, version 21.0. The Kolmogorov–Smirnov test was first applied to assess the data distribution's normality. Continuous variables were expressed as mean ± standard deviation (SD) or median with interquartile range (IQR). Student t-tests or variance analyses (ANOVA) were used for normally distributed data to compare means between groups. Medians were compared using the Mann–Whitney U and Kruskal-Wallis tests for non-normally distributed data. Correlation coefficients between continuous variables were calculated using the Spearman rank order test and the Pearson correlation test. Frequencies were compared using Pearson and Fisher's exact test. All results were analyzed with a 95% confidence interval, and statistical significance was defined as a *p*-value < 0.05. The required sample size was 114 for a two-tailed t-test with a significance level of 5% to achieve a statistical power of 84%.

## 3 RESULTS

### 3.1 Participants’ Characteristics

The study flow chart is shown in Fig. (**1**). A total of 58 primary glomerulonephritis patients and 58 healthy controls were enrolled in this study. The patients had a mean age of 44.6 ± 15.3 years, with 24 (41.4%) females. In the healthy control group, the average age was 43.9 ± 9.4 years, with 22 (37.9%) females. No significant differences were observed between the two groups regarding sex and age (*p* = 0.993 and *p* = 0.840, respectively). The general characteristics of both patients and healthy controls are summarized in Table **1**.

According to the renal biopsy findings, the most prevalent form of glomerulonephritis was immunoglobulin A (IgA) nephritis, accounting for 32.8% (*n* = 19) of cases. This was followed by focal segmental glomerulosclerosis (FSGS) at 29.3% (*n* = 17), membranous glomerulonephritis at 22.4% (*n* = 13), minimal change disease at 10.3% (*n* = 6), membranoproliferative glomerulonephritis at 3.4% (*n* = 2), and postinfectious glomerulonephritis at 1.7% (*n* = 1).

Among the patients, 41.4% (*n* = 24) had hypertension, 41.4% (*n* = 24) had hyperlipidemia, 13.8% (*n* = 8) had diabetes, 6.9% (*n* = 4) had coronary artery disease, 6.9% (*n* = 4) had osteoporosis, and 5.2% (*n* = 3) had heart failure. Renin-angiotensin aldosterone blockers were used by 43 (71.7%) of the patients, and 32 (55.2%) received immunosuppressive drugs.

### 3.2 Clinical Findings

The frequency of subclinical hypothyroidism detected in primary glomerulonephritis patients was 17.2% (*n* = 10), and overt hypothyroidism was 8.6% (*n* = 5). Overt hypothyroidism was not detected in any of the healthy controls, while 3.4% (*n* = 2) had subclinical hypothyroidism. The frequency of anti-TPO positivity in patients with primary glomerulonephritis was 10.3% (*n* = 6), and anti-TG positivity was 6.9% (*n* = 4). Both anti-TPO and anti-TG were positive in 8.3% (*n* = 5) of healthy controls.

### 3.3 Biochemical and Sonographic Findings

The laboratory data of the participants are presented in Tables **2** and **3**. Notably, there were no statistically significant differences observed between the patients and healthy controls in terms of TSH levels (*p* = 0.874), FT4 levels (*p* = 0.818), anti-TPO levels (*p* = 0.668), and anti-TG levels (*p* = 0.096). However, the FT3 levels in the patient group (2.80 ± 0.78 pg/ml) were significantly lower than those in the healthy control group (3.29 ± 0.45 pg/ml, *p* < 0.001).

Table **4** compares the thyroid ultrasonography findings between patients with primary glomerulonephritis and healthy controls. Notably, the thyroid gland structure in primary glomerulonephritis patients exhibited more hypoechoic and heterogenous than healthy controls. Furthermore, the prevalence of nodular goiter was notably higher among the primary glomerulonephritis patients.

### 3.4 Comparison of Thyroid Examination According to the Use of Immunosuppressive Therapy

At the time of evaluation, 32 patients (55.2%) underwent immunosuppressive therapy. Table **5** compares thyroid examination findings between patients using and not using immunosuppressive treatment. No significant differences were observed in TSH, FT4, FT3, anti-TPO, and anti-TG levels between patients using immunosuppressive therapy and those who did not use it.

### 3.5 Relationship Between TSH Level and Kidney Function Tests

The correlation analysis between TSH levels and kidney function tests in patients with primary glomerulonephritis is presented in Table **6**. Among the parameters included in the analysis, a linear relationship was found only between 24-hour urinary albumin levels, 24-hour urinary protein levels, and TSH (*p* = 0.049 and *p* = 0.044, respectively).

## 4 DISCUSSION

This study reported that the prevalence of subclinical and overt hypothyroidism increased in patients with primary glomerulonephritis compared to healthy controls. 24-hour urine albumin and 24-hour urine protein levels showed a linear relationship with TSH levels in patients with primary glomerulonephritis. The most prominent thyroid biochemical abnormality in patients with primary glomerulonephritis was a low FT3 level. The incidence of Hashimoto's thyroiditis was similar to that of healthy controls. On sonographic examination, goiter, thyroid nodules, heterogeneous glandular structure, low gland echogenicity, and pseudonodular hypoechoic infiltration were significantly more common in patients with primary glomerulonephritis than healthy controls.

In this study, we found an increased prevalence of subclinical and overt hypothyroidism in patients with primary glomerulonephritis. Many studies have reported a higher incidence of thyroid dysfunction in patients with CKD. Hypothyroidism and subclinical hypothyroidism are the most commonly observed changes [16-19]. Changes in serum thyroid hormone levels have been found in diseases that lead to leakage of plasma proteins into the urine and impair glomerular barrier function [20-23]. The loss of proteins in the urine that bind thyroid hormones, such as thyroxine-binding globulin, transthyretin, prealbumin, and albumin, can lead to a decrease in serum total thyroxine and occasionally total triiodothyronine levels. These hormonal changes depend on the extent of proteinuria and reduced serum albumin levels [20]. Patients with free T4 and T3 levels below the normal range may remain euthyroid because the thyroid gland compensates for these hormone losses in the urine by increasing production. However, overt hypothyroidism may develop in patients with low thyroid reserve [22]. In this context, our results may explain the higher prevalence of subclinical and overt hypothyroidism in patients with primary glomerulonephritis, a group of patients with CKD who have proteinuria, compared to healthy controls.

Case reports in the literature suggest an association between hypothyroidism and nephrotic syndrome, and a small study on 60 patients showed a relationship between TSH levels and the degree of proteinuria [24]. Another study reported a sixfold higher prevalence of subclinical hypothyroidism in 159 patients with proteinuria compared to 900 controls [25]. Kwong *et al.* confirmed the high prevalence of hypothyroidism in a large cohort of patients with proteinuria. They showed that the risk of hypothyroidism increased with the severity of proteinuria [26]. Primary glomerulonephritis is commonly characterized by the presence of proteinuria. Although albumin accounts for the majority of protein excreted because of the selective nature of proteinuria, many other essential globulins, hormones, and hormone-binding proteins are also excreted in significant amounts, leading to clinical consequences unrelated to renal dysfunction per se. Urinary T4 and thyroid-binding globulin (TBG) losses are an important concern. Although metabolic consequences are not expected since FT3 and FT4 levels remain normal in the early stages, prolonged excretion of TBG may reduce free thyroid hormone levels, which, in turn, leads to an increase in TSH levels as the thyroid gland becomes overactive to maintain constant hormone levels [1, 2]. Our study also found that TSH levels increased with increasing proteinuria. Our findings are consistent with studies conducted in other CKD patients and have been demonstrated for the first time in patients with primary glomerulonephritis. Our study did not detect the inverse relationship between GFR and TSH reported in the literature [24-26]. This may be due to our study population's slightly lower GFR (mean 83.75 mL/min/1.73 m^2^). It should be confirmed by further studies with more extensive patient series.

The earliest and most common thyroid dysfunction in CKD patients is a low T3 level [27]. This “low T3 syndrome” occurs in CKD for various reasons. Starvation, chronic metabolic acidosis, and a chronic low-protein diet impair T3 protein binding and iodothyronine diodination, reducing peripheral conversion of T4 to T3 and protein binding. In addition, inflammatory cytokines, such as tumor necrosis factor (TNF)-a and interleukin (IL)-1, inhibit the expression of type 1 5'-deiodinase, which is responsible for the peripheral conversion of T4 to T3 [28]. In addition, restricted renal iodine intake increases serum iodine levels and prolongs the Wolff-Chaikoff effect [29]. In this study, FT3 levels were significantly lower in patients with primary glomerulonephritis than healthy controls. In patients with kidney disease, FT4 levels are low to average. This is primarily due to impaired protein binding of T4. When total T4 is measured in patients, the T4 level is often low, but the FT4 level is usually expected in these patients. The role of T4 and TSH in morbidity and mortality in renal patients appears to be less than that of T3 [30]. This study showed that FT4 levels were similar in patients with primary glomerulonephritis and healthy controls.

Although autoimmune thyroid disease has occasionally been reported in patients with glomerulonephritis, a causal relationship between the two conditions has not been established [31-33]. Furthermore, an increased incidence of autoimmune thyroiditis in CKD patients has not been reported [34]. This study found that autoimmune thyroiditis was not higher in patients with primary glomerulonephritis than in healthy controls. These results are consistent with the literature. The presence of immunosuppressive drugs, which could affect antibody positivity, was investigated for autoimmune disease, and there was no difference between patients taking medication and those who did not.

This study found that the thyroid volume of patients with primary glomerulonephritis is significantly higher than healthy controls. CKD leads to an increase in serum inorganic iodide levels and a decrease in iodide excretion, which increases the iodine content of the thyroid gland and consequently leads to an enlargement of the thyroid gland. Patients with chronic kidney disease have low T3 and normal or low T4 levels, which leads to an increase in TSH and, consequently, to an enlargement of the thyroid gland [34, 35]. These factors may explain the goiter observed in patients with primary glomerulonephritis.

Decreased echogenicity in the thyroid gland can be attributed to various factors, including decreased colloid content, increased blood flow to the thyroid gland, or increased lymphocytic infiltration [36-38]. The classification of echogenicity into three categories is a modification of the classification system proposed initially by Sostre and Reyes [39]. This study found a higher prevalence of hypoechogenic structures of the thyroid parenchyma in patients with primary glomerulonephritis compared to healthy controls. The increased hypoechogenicity found in patients with primary glomerulonephritis suggests increased inflammatory activity in the thyroid gland independent of thyroid antibodies. In addition, this study evaluated another sonographic variable, echotexture, which was categorized as either homogeneous or heterogeneous. A heterogeneous internal structure was found in 60.3% of patients. This heterogeneous echotexture was considered an indicator of increased inflammatory activity in the thyroid gland in these patients. In addition, hypoechoic pseudonodular and multifocal infiltrates, representing regions of intense inflammatory activity (*e.g.,* lymphocytic infiltration), were found in 20.7% of patients. This rate was significantly higher than in healthy controls.

Lin *et al.* reported an increased prevalence of thyroid nodules in their study on 221 patients with ESRD [40]. Another study highlighted the increased frequency of thyroid nodules in CKD patients and emphasized the importance of regular sonographic examinations [41]. Sanai *et al.* reported an increased prevalence of thyroid nodules in CKD hemodialysis patients [42]. However, until now, there has been no information on the prevalence of thyroid nodules in patients with primary glomerulonephritis. This study is the first to report that the prevalence of thyroid nodules is increased in patients with primary glomerulonephritis compared to healthy controls.

Many gaps in our knowledge of the interaction between thyroid and kidney disease still need to be filled. Further studies are required to understand how kidney disease can lead to thyroid dysfunction. More sensitive and specific methods of classifying thyroid functional status in renal patients need to be found to more accurately distinguish actual functional thyroid disease from non-thyroid disease and physiologic adaptation in this population. Careful and systematic sonographic examination may be helpful in the diagnosis and treatment decision of thyroid disease in this population.

## 5 LIMITATIONS

This study has some limitations. Due to its cross-sectional design, it was impossible to investigate the relationship between disease progression and thyroid status. The disease was rare, so the study was conducted with a relatively small number of patients. Furthermore, the causal relationship between thyroid disorders and primary glomerulonephritis could not be proven by pathological examination.

## CONCLUSION

The prevalence of overt and subclinical hypothyroidism was found to be increased in patients with primary glomerulonephritis compared to the healthy population. A linear correlation was found between the level of proteinuria and the TSH value. In addition, the incidence of nodular goiter was higher in these patients than in healthy controls. As primary glomerulonephritis mainly occurs in young people and these patients suffer from chronic kidney disease for life, we emphasize the importance of regular thyroid function tests and sonographic examinations for thyroid disorders.

## Figures and Tables

**Fig. (1):**
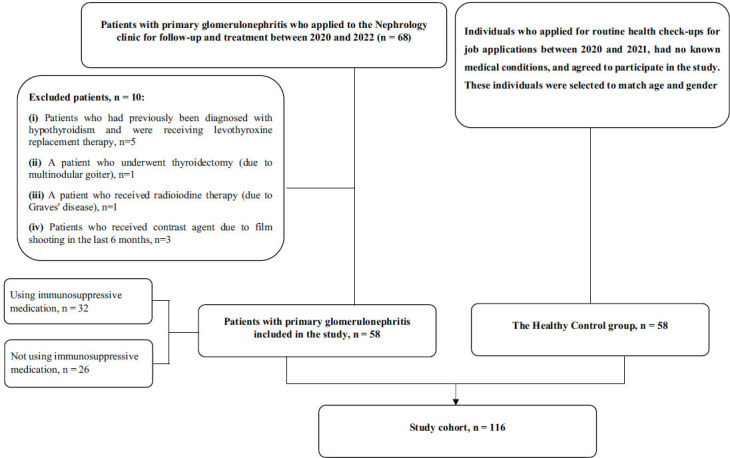
Study flow chart.

**Table 1:** General characteristics of participants.

**Characteristics**	**Patients with Primary Glomerulonephritis (*n* = 58)**	**Healthy Controls (*n* = 58)**	***p*-Value**
Age (year), mean ± SD	44.6 ± 15.3	43.9 ± 9.4	0.993
Sex, female, n (%)	24 (41.4)	22 (37.9)	0.840
Smoking, n (%)	23 (38.3)	13 (22.4)	0.033
**Blood pressure, mean ± SD**			
Systolic (mmHg)	126.3 ± 13.2	118.9 ± 10.9	< 0.001
Diastolic (mmHg)	78.5 ± 11.9	78.3 ± 8.2	0.482
Heart rate (/min), mean ± SD	75.9 ± 6.6	74.4 ± 6.2	0.224
BMI (kg/m^2^), mean ± SD	26.4 ± 4.1	26.8 ± 4.5	0.593

**Abbreviation:** BMI, Body mass index; SD, Standard deviation

**Table 2:** Comparison of participants' biochemical test findings.

**Biochemical Tests**	**Patients with Primary Glomerulonephritis (*n* = 58)**	**Healthy Controls (*n* = 58)**	***p*-Value**
WBC (10^3^/μL), NV 4.3-10.3, mean ± SD	9.3 ± 3.2	7.0 ± 1.9	< 0.001
Hemoglobin (g/dL), NV 13.6-17.2, mean ± SD	12.83 ± 2.17	13.62 ± 1.27	0.019
Platelet (10^6^/μL), NV 156-373 median (IQR)	292.0 (249.3–335.0)	260.0 (214.5–304.3)	0.098
Sodium (mmol/L), NV 136-145, mean ± SD	140.29 ± 2.96	137.53 ± 18.52	0.928
Potassium (mmol/L), NV 3.5-5.1, mean ± SD	4.49 ± 0.51	4.25 ± 0.65	0.032
AST (IU/L), NV <40, mean ± SD	19.54 ± 9.16	18.66 ± 4.34	0.260
ALT (IU/L), NV <41, mean ± SD	22.01 ± 15.15	18.37 ± 7.28	0.674
Albumin (g/dL), NV 3.5-5.2, mean ± SD	3.83 ± 0.90	4.41 ± 0.89	0.001
Calcium (mg/dL), NV 8.4-10.2, mean ± SD	9.01 ± 0.69	9.25 ± 0.38	0.147
Phosphor (mg/dL), NV 2.5-4.5, mean ± SD	3.68 ± 0.75	3.12 ± 1.28	0.087
Parathormone (ng/L), NV 15-65, median (IQR)	32.10 (21.50–47.45)	30.10 (3.45–42.10)	0.249
Uric acid (mg/dL), NV 3.4-7, median (IQR)	5.95 (4.98–7.40)	4.05 (3.48–4.93)	< 0.001
25-OH-D (μg/L), NV ≥30, mean ± SD	17.42 ± 10.47	21.57 ± 10.96	0.039
CRP (mg/L), NV <5, median (IQR)	1.66 (0.68–3.78)	1.63 (0.91–2.99)	0.908
LDL-C (mg/dL), NV <100, mean ± SD	151.75 ± 72.74	132.33 ± 32.90	0.449
HDL-C (mg/dL), NV >40, mean ± SD	55.65 ± 20.77	55.14 ± 15.63	0.634
Triglyceride (mg/dL), NV <200, mean ± SD	192.91 ± 92.67	116.69 ± 67.06	< 0.001

**Abbreviations:** ALT, alanine aminotransferase; AST, aspartate aminotransferase; CRP, c-reactive protein; HDL, high-density lipoprotein; IQR, interquartile range; LDL, low-density lipoprotein; NV, normal values; OH-D, hydroxy vitamin D; SD, standard deviation; WBC, white blood cell.

**Table 3:** Comparison of participants' thyroid and kidney function test findings.

**Tests**	**Patients with Primary Glomerulonephritis (*n* = 58)**	**Healthy Controls (*n* = 58)**	***p*-Value**
TSH (μIU/mL), NV 0.27-4.2, median (IQR)	1.96 (1.08–3.91)	1.72 (1.23–2.73)	0.874
FT4 (ng/dL), NV 0.93-1.7, mean ± SD	1.04 ± 0.30	1.21 ± 0.17	0.818
FT3 (pg/mL), NV 2-4.4, mean ± SD	2.80 ± 0.78	3.29 ± 0.45	< 0.001
TPO-Ab (IU/mL), NV 0-34, median (IQR)	10.72 (7.34–17.90)	13.05 (8.56–17.80)	0.668
TG-Ab (IU/mL), NV 0-115, median (IQR)	13.72 (11.70–17.80)	12.93 (10.0–18.15)	0.096
Urea (mg/dL), NV 17-49, mean ± SD	48.79 ± 33.44	23.59 ± 7.21	0.041
Creatinine (mg/dL), NV 0.7-1.2, mean ± SD	3.43 ± 1.6	0.72 ± 0.19	0.040
eGFR (mL/min/1.73 m^2^), NV >90, mean ± SD	83.75 ± 38.26	105.0 ± 18.3	< 0.001
24-h urine protein (g/day), NV <0.15, median (IQR)	2.86 (0.79–5.69)	-	-
24-h urine albumin (g/day), NV <0.03, median (IQR)	2.15 (0.62–4.58)	-	-
Hematuria, n (%)	35 (60.3)	4 (6.9)	< 0.001
Leukocyturia, n (%)	16 (27.6)	3 (5.2)	< 0.001

**Abbreviations:** eGFR, estimated glomerular filtration rate; FT3: free triiodothyronine; FT4, free thyroxine; NV, normal values; SD, standard deviation; TG-Ab, thyroglobulin antibody; TPO-Ab, thyroid peroxidase antibody; TSH, thyroid stimulating hormone.

**Table 4:** Comparison of thyroid ultrasound findings.

**Ultrasound Findings**	**Patients with Primary Glomerulonephritis (*n* = 58)**	**Healthy Controls (*n* = 58)**	***p*-Value**
Volume in cubic centimeters (cm^3^), mean ± SD	12.69 ± 5.67	8.95 ± 5.20	< 0.001
**Echogenicity, n (%)**			< 0.001
E1-isoechoic	27 (46.6)	44 (75.9)	
E2-mildly hypoechoic	20 (34.5)	13 (22.4)	
E3-hypoechoic	11 (19)	1 (1.7)	
**Sonographic texture, n (%)**			< 0.001
S1-homogeneous	23 (39.7)	45 (77.6)	
S2-heterogeneous	35 (60.3)	13 (22.4)	
**Pseudonodular hypoechoic infiltration, n (%)**			0.050
I0-no	46 (79.3)	50 (86.2)	
I1-yes	12 (20.7)	8 (13.8)	
**Nodules, n (%)**			< 0.001
N0-no	38 (65.5)	56 (96.6)	
N1-yes	20 (34.5)	2 (3.4)	
**Cysts, n (%)**			0.382
C0-no	53 (91.4)	50 (86.2)	
C1-yes	5 (8.6)	8 (13.8)	

**Abbreviation:** SD, Standard deviation.

**Table 5:** Comparison of thyroid function tests and sonography of those who received and did not receive immunosuppressive therapy.

**Parameters**	**Immunosuppressive Drug Use**		***p*-Value**
	**Yes (*n* = 32)**	**No (*n* = 26)**	
TSH (μIU/mL), NV 0.27-4.2, median (IQR)	1.59 (0.78 – 3.82)	2.02 (1.33 – 4.05)	0.089
FT4 (ng/dL), NV 0.93-1.7, mean ± SD	1.18 ± 0.35	1.19 ± 0.16	0.088
FT3 (pg/mL), NV 2-4.4, mean ± SD	2.75 ± 0.82	2.84 ± 0.67	0.208
TPO-Ab (IU/mL), NV 0-34, median (IQR)	11.03 (6.85 – 19.88)	10.72 (7.55 – 16.23)	0.259
TG-Ab (IU/mL), NV 0-115, median (IQR)	13.67 (11.55 – 17.23)	13.72 (11.75 – 19.25)	0.194
Volume in cubic centimeters (cm^3^), mean ± SD	13.12 ± 6.60	12.15 ± 4.32	0.061
**Echogenicity, n (%)**			0.994
E1-isoechoic	15 (46.9)	12 (46.2)	
E2-mildly hypoechoic	11 (34.4)	9 (34.6)	
E3-hypoechoic	6 (18.8)	5 (19.2)	
**Sonographic texture, n (%)**			0.742
S1-homogeneous	13 (40.6)	10 (38.5)	
S2-heterogeneous	19 (59.4)	16 (61.5)	
**Pseudonodular hypoechoic infiltration, n (%)**			0.431
I0-no	26 (81.3)	20 (76.9)	
I1-yes	6 (18.7)	6 (23.1)	
**Nodules, n (%)**			0.103
N0-no	18 (56.3)	20 (76.9)	
N1-yes	14 (43.7)	6 (23.1)	
**Cysts, n (%)**			0.022
C0-no	32 (100)	21 (80.8)	
C1-yes	0 (0)	5 (19.2)	

**Abbreviations:** FT3, free triiodothyronine; FT4, free thyroxine; NV, normal values; IQR, interquartile range; SD, standard deviation; TG-Ab, thyroglobulin antibody; TPO-Ab, thyroid peroxidase antibody; TSH, thyroid stimulating hormone.

**Table 6:** Correlation of TSH level and kidney function tests in patients with primer glomerulonephritis.

**Kidney Function Tests**	**TSH (μIU/mL)**	
	***p*-Value**	**r-Value**
Urea (mg/dL)	0.613	0.071
Creatinine (mg/dL)	0.862	0.024
eGFR (mL/min/1.73 m^2^)	0.478	-0.100
24-hour urine albumin (g/day)	0.049	0.272
24-hour urine protein (g/day)	0.044	0.277

**Abbreviations:** eGFR, estimated Glomerular filtration rate; TSH, thyroid stimulating hormone; R-value, correlation coefficient.

## Data Availability

This article and its tables include all data obtained or analyzed as part of this study. The data archive can be made available on request. Further requests can be directed to the corresponding author.

## LIST OF ABBREVIATIONS

CKD: Chronic Kidney Disease
CRP: C-reactive Protein
GFR: Glomerular Filtration Rate
HC: Healthy Controls
HDL: High-Density Lipoprotein
LDL: Low-Density Lipoprotein
TG: Triglycerides
TSH: Thyroid-Stimulating Hormone
SD: Standard Deviation

## STANDARDS OF REPORTING

STROBE guidelines were followed.
